# Supplementary material for: Trans and gender diverse people’s experiences of healthcare access in Australia: A qualitative study in people with complex needs
Source: PLoS One. 2021 Jan 28;16(1):e0245889. doi: 10.1371/journal.pone.0245889 (PMC7842963; doi:10.1371/journal.pone.0245889)
Supplement: Attachment — Submitted filename: Response to reviewers trans paper.docx [file pone.0245889.s001.docx]

Thanks too the reviewers for their thoughtful and detailed recommendations.

Reviewer #1

1) I don’t know that the first sentence is correct in terms of being understudied: of all the research in the field, research on health and the impact of discrimination likely constitutes the bulk of research

*We have changed the first sentence to:*

*The global disease and health burden of trans and gender diverse (TGD) people are poorly understood, and the impacts of stigma, discrimination and violence are particularly serious.*

2) The paper switches between writing out, for example, trans woman as two words, and combining them as one. Having it uniform as two words throughout would be preferable

*Thank you for this observation – we have identified all instances of the combining into one word, and changed to two words.*

3) Not sure that AMAB and AFAB are necessary in the table?

*Our research team had been ambivalent about the inclusion of AMAB and AFAB, and we are very happy to take reviewer 1’s advice on this. These have now been removed (Table 1, pp8-10)*

4) The following chapter might be of interest to the authors given the focus on complexity of care: Riggs, D.W., & Bartholomaeus, C. (2017). The disability and diagnosis nexus: Transgender men navigating mental health care services. In C. Loeser, V. Crowley & B. Pini (Eds.) Disability and masculinities: Corporeality, pedagogy, and the critique of otherness (pp. 67-84). London: Palgrave.

*Thank you for recommending this chapter. We have included a reference to it on page 4.*

*This ‘gateway’ diagnosis is problematic not only because it is implicitly pathologizing, but because it may render a TGD person’s mental health issues invisible in contexts where accessing gender affirming treatment may take priority over full and frank discussion of mental health with a clinician.(10)*

*We have also removed the word ‘comorbidities’ in the manuscript, in response to the critique of that term made in this chapter(see line 546).*

5) I think actual pseudonyms would be preferable to initials. Also, the method doesn’t state if these initials are pseudonyms

*We have changed all initials to pseudonyms and noted this in the text (lines 166 and 180).*

6) The results sit on the side of a 'extract and comment' approach, which makes for a rather thin analysis of the data. Ideally, a little more detailed analysis of each extract would be undertaken: as per Braun and Clarke, a focus on latent as well as semantic meaning. This might mean reducing the number of extracts in each sub theme as at present they are rather long

We thank the reviewer for this comment and acknowledge that there is a descriptive emphasis in this study. The research team wants to keep the extracts in the results section relatively long so a sense of the voices of the participants can be discerned in the text.

7) Some of the themes or sub themes have specific titles when the extracts are actually quite broad. The mental health sub theme, for example, includes extracts that have seemingly little to do with mental health. Either the contents of the themes needs to be clarified, or a more indicative title used. Reducing the number of extracts as per 6 might also help with this.

In the originally submitted version mental health was specifically addressed in the subtheme, *Complexity in mental healthcare (lines 229-261). In response to the reviewer, we have renamed this subtheme ‘mental healthcare’. The issue of cost as a barrier to all forms of healthcare, including mental health, was then addressed under the theme ‘affording healthcare’ under the subtheme ‘economic hardship’. In this subtheme, the cost of mental healthcare is discussed alongside costs of dental healthcare and costs of prescription medication. We think that this is well justified because costs of healthcare was a significant issue for all participants, and this was not limited to mental health. Keeping this theme separate allows the reader to see the catastrophic impacts of delaying dental over time, with the example of Jen losing all her teeth and also bone mass due to her long term economic deprivation.*

8) The sub theme title 'attributes' is not descriptive (ie it doesn’t describe the theme contents)

*We agree with the reviewer, and have changed the name of this subtheme to ‘positive qualities’.*

Reviewer #2

This article contributes to a small but important body of knowledge about the health care needs of a hard to reach group. The authors acknowledge that it is a small sample and not representative in any sense, nevertheless the data collected is valuable in informing reform of the health system.

The study is well designed and the methodology sound. The literature is adequate for the scale of the study. The research has clearly been carried out with a great deal of cultural sensitivity and so serves to demonstrate how data can be collected from those who are disenfranchised and so often not heard.

The data is used to shape practical suggestions about health care provision in the future for those with complex needs. It makes a valuable contribution to the body of knowledge in relation to transgender people.

*We thank the reviewer for her supportive comments.*
